# Quality assessment of platelet concentrates prepared by platelet rich plasma-platelet concentrate, buffy coat poor-platelet concentrate (BC-PC) and apheresis-PC methods

**DOI:** 10.4103/0973-6247.53882

**Published:** 2009-07

**Authors:** Ravindra P. Singh, Neelam Marwaha, Pankaj Malhotra, Sumitra Dash

**Affiliations:** *Deputy of Research, Kurdistan University of Medical Sciences, Iran*; 1 *Sanandaj District Health Center, Kurdistan University of Medical Sciences, Iran*; 2 *Kurdistan Organization of Blood Transfusion –Research Center, Iran*; 3 *Iranian Organization of Blood Transfusion –Research Center Iran*

**Keywords:** Corrected count increment, buffy coat poor-platelet concentrate, percentage recovery, platelet concentrate, platelet rich plasma-platelet concentrate, random donor platelet, single donor platelets

## Abstract

**Background:**

Platelet rich plasma-platelet concentrate (PRP-PC), buffy coat poor-platelet concentrate (BC-PC), and apheresis-PC were prepared and their quality parameters were assessed.

**Study Design:**

In this study, the following platelet products were prepared: from random donor platelets (i) platelet rich plasma - platelet concentrate (PRP-PC), and (ii) buffy coat poor-platelet concentrate (BC-PC) and (iii) single donor platelets (apheresis-PC) by different methods. Their quality was assessed using the following parameters: swirling, volume of the platelet concentrate, platelet count, WBC count and pH.

**Results:**

A total of 146 platelet concentrates (64 of PRP-PC, 62 of BC-PC and 20 of apheresis-PC) were enrolled in this study. The mean volume of PRP-PC, BC-PC and apheresis-PC was 62.30±22.68 ml, 68.81±22.95 ml and 214.05±9.91 ml and ranged from 22-135 ml, 32-133 ml and 200-251 ml respectively. The mean platelet count of PRP-PC, BC-PC and apheresis-PC was 7.6±2.97 × 1010/unit, 7.3±2.98 × 1010/unit and 4.13±1.32 × 1011/unit and ranged from 3.2 –16.2 × 1010/unit, 0.6-16.4 × 1010/unit and 1.22-8.9 × 1011/unit respectively. The mean WBC count in PRP-PC (n = 10), BC-PC (n = 10) and apheresis-PC (n = 6) units was 4.05±0.48 × 107/unit, 2.08±0.39 × 107/unit and 4.8±0.8 × 106/unit and ranged from 3.4 -4.77 × 107/unit, 1.6-2.7 × 107/unit and 3.2 – 5.2 × 106/unit respectively. A total of 26 units were analyzed for pH changes. Out of these units, 10 each were PRP-PC and BC-PC and 6 units were apheresis-PC. Their mean pH was 6.7±0.26 (mean±SD) and ranged from 6.5 – 7.0 and no difference was observed among all three types of platelet concentrate.

**Conclusion:**

PRP-PC and BC-PC units were comparable in terms of swirling, platelet count per unit and pH. As expected, we found WBC contamination to be less in BC-PC than PRP-PC units. Variation in volume was more in BC-PC than PRP-PC units and this suggests that further standardization is required for preparation of BC-PC. As compared to the above two platelet concentrates, all the units of apheresis-PC fulfilled the desired quality control criteria of volume. Apheresis-PC units showed better swirling and platelet count than PRP-PCs and BC-PCs. All the platelet concentrates units had pH well above the recommended norm.

## Introduction

Since platelets were first identified in 1881, there has been continuous and accelerating progress in our basic understanding of platelet function[1] and its utilization in various bleeding disorders.The first successful attempt to raise the platelet count in thrombocytopenic patients by transfusion of whole blood was described by Duke in 1910. General improvement of the technique to separate platelets from whole blood and availability of plastic bags in blood banking revolutionized the field of component therapy. Platelet transfusions are the primary therapy for thrombocytopenia due to various causes. Thrombocytopenia may be due to qualitative defect, i.e. defect in platelet function or quantitative defect, i.e. decreased platelet count which can be seen in various hemato-oncological patients either due to primary disease or chemotherapy.

Two types of platelet concentrates are available for transfusion; one which is the co-product of normal blood donation i.e. random donor platelets (RDP), (platelet rich plasma-platelet concentrate (PRP-PC) and buffy coat poor-platelet concentrate (BC-PC) and the other is single donor platelets (SDPs), (apheresis-PC,) collected from voluntary thrombocytapheresis donors with the help of an automated cell separator. The basic principle behind preparation of components from whole blood is that each component has its specific gravity and by applying centrifugation, each component is separated and removed, thus allowing the transfusion of desired component according to the need of the patient. The recommended shelf life of platelet concentrates in presently available platelet storage bags is 5 days at 22±2°C with continuous agitation. The platelets undergo various storage changes starting from collection, processing to storage and the underlying conditions within the patients, which may affect the therapeutic benefit to the recipient.[2]

The *in vitro* platelet quality can be assessed by using certain parameters (swirling, volume, platelet count and WBC count per bag and pH changes) and in vivo by using corrected count increment (CCI) and percentage recovery (PR) at 1 hour and 20 hours post transfusion which accesses the functional platelets in circulation. If the CCI at 1 hr and 20 hours is < 7500 platelets/µL/m^2^ and < 4500 platelet/µL/m^2^ and PR at 1 hour and 20 hours <30% and 20% respectively on two consecutive occasions it indicates platelet transfusion refractoriness.

In this study we have analyzed the quality of different platelet concentrates prepared by different methods as per the recommended quality norms.[3] [Table 1]

**Table 1:** Criteria for quality assessment of PRP-PC, BC-PC and apheresis-PC (DGHS, India recommendation)[3]

Parameters	Frequency of control	Quality requirement for PRP-PC and BC-PC	Quality requirement for apheresis-PC
Swirling	All units	Present	Present
Volume	All units	40-70 ml (PRP-PC), 70-90 ml (BC-PC)	200-300 ml
Platelet count/ unit	Monthly	>5.5 × 1010 in 75% of unit tested	>3×1011 in 75% of units tested
WBC count/unit	Monthly	5.5 × 107 – 5 × 108	<5 × 106 in each unit tested and post leukoreduction retains a minimum of 85% of original platelets in the component.
pH	Monthly	>6.0 at the end of maximum days of storage in 100% of units tested.	>6.0 at the end of maximum days of storage in 100% of units tested.

## Materials and Methods

In this study, the following platelet products were prepared; random donor platelets (i) platelet rich plasma - platelet concentrate (PRP-PC), and (ii) buffy coat poor - platelet concentrate (BC-PC) and (iii) single donor platelets (apheresis-PC) by different methods. Their quality was assessed using the following parameters: swirling, volume of the platelet concentrate, platelet count, WBC count and pH. This study was conducted in the departments of Transfusion Medicine of the institute. The samples were collected between January 2003 and December 2003. During this period, a total of 44,069 whole blood units were collected, and 7,167 random donor platelets [PRP-PC-3,081(6.9%) and BC-PC-810(1.8%)] were prepared.

Single donor platelets were prepared from 98 donors by apheresis.

### Random donor platelets

Whole blood was collected in 450-ml bags containing 63 ml of CPDA1 anticoagulant, kept at room temperature (20-24°C) and PRP-PC was prepared within 4 hours of collection and turning the whole blood upside down several times to mix the contents before centrifugation.

The donor arm was selected and blood pressure raised by a BP cuff to locate and select good vein for phlebotomy. After the selection of a suitable vein, the donor arm was prepared by cleaning venipuncture sites at antecubital area starting from center to periphery of the selected arm by betadine and spirit without contamination of selected phlebotomy site, then the phlebotomy was done with minimal trauma and BP set between 40-60 mmHg with continuous pressing of the sponge ball by the same hand to maintain good blood flow. The blood collection was completed within 5±3 minutes from the starting time. The blood flow rate was maintained between 50-70±5 ml/minute with continuous mixing at the rate of 45±10 second. None of the collected blood units exceeded total collection time of 10 minutes.

### Method of preparation of platelet rich plasma – platelet concentrate (PRP-PC)

Four hundred and fifty ml of whole blood was collected in a 450-ml triple bag containing CPDA1 anticoagulant (TERUMO PENPOL, Ltd. Puliyarakonam, Trivandrum, India). Platelet rich plasma was separated from whole blood by light spin centrifugation by Heraeus 6000i, Germany refrigerated centrifuge at 1750 rpm for 11 minutes at 21°C, with acceleration and deceleration curves of 5 and 4 respectively and the platelets were concentrated by heavy spin centrifugation at 3940 rpm for 5 minute at 21°C, with acceleration and deceleration curves of 9 and 5 respectively with subsequent removal of supernatant plasma. The platelet concentrate bag was left stationary with the label side down at room temperature for approximately 1 hour. The platelet poor plasma was frozen promptly and stored as fresh frozen plasma (FFP) at or below -30°C for one year.

### Method of preparation of buffy coat - platelet concentrate (BC-PC)

Four hundred and fifty ml of whole blood was collected in a 450-ml quadruple bag containing 63 ml of CPD anticoagulant, with additive solution (SAGM). (TERUMO PENPOL, Ltd., Puliyarakonam, Trivandrum, India). The whole blood was first subjected to “hard spin” centrifugation at 3940 rpm for 5 minutes at 21°C with acceleration and deceleration curves of 9 and 4 respectively. Whole blood was separated into different components according to their specific gravity.

- The top layer – platelet poor supernatant plasma (150-200 ml).
- Middle layer – buffy coat, containing approximately 90% of platelets, 70% of WBCs and 10% of red cells.
- Bottom layer – packed red cells.

Platelet poor supernatant was expressed into one satellite bag and buffy coat into another satellite bag. About 20-30 ml of plasma was returned to buffy coat with the aim of cleaning the tubing from residual cells and obtaining an appropriate amount of plasma in the BC. The SAGM solution was added to the red cells. The bags containing red cells and plasma were then removed. Red cells were placed at 4°C in cold room and platelet poor plasma into a -40°C deep freezer as fresh frozen plasma (FFP). The buffy coat was gently mixed with the plasma and again subjected to ‘light spin" centrifugation at 1,100 rpm for 6 minutes at 21°C, with acceleration and deceleration curves of 5 and 4 respectively, along with one empty satellite bag. The supernatant, platelet rich plasma was expressed into empty platelet storage bag and then tubing was sealed. The bag with residual WBCs and red cells was discarded.

### Single donor platelets (SDP)-apheresis-PC

The automated cell separator equipment may be of intermittent flow or continuous flow cell technique, using single or double venous access. We used continuous flow, double venous access, automated cell separator – CS3000 ® plus, Baxter, Fenwal division, Deerfield, 14 60015, USA.

1. Written consent of the donor was taken after explaining the procedure in detail, time taken and about possible hazards and benefits.
2. Venous access is an important consideration in apheresis donors and veins were examined at the time of selection because of the following reasons -
  - Long duration of procedure
  - Prolonged flow rate
  - Frequent need for two venipunctures with continuous flow equipment.
3. Age of the donors was between 18-50 years.
4. Prior to the apheresis procedure, ABO / Rh typing and testing for infections disease markers (HIV, HBV, HCV, VDRL and malaria) of plateletpheresis-donors were done.
5. Donors who had taken aspirin or other NSAIDS, which are likely to affect the platelet function, were deferred.
6. Those donors who had platelet count > 1.5 × 10^5^/µl were taken for plateletpheresis.

### Procedure

The procedure was done in a closed system. A disposable kit was installed on to the continuous flow separator, then the machine was primed. The donor was prepared by cleaning two venipuncture sites at antecubital area of both arms by betadine and spirit, and then phlebotomy was done with minimal trauma to the donor. During the procedure, the blood was anti-coagulated at the point of withdrawal in a controlled manner, and the ratio of whole blood and anticoagulant (ACD) was maintained at 9:1 to 11:1. The anticoagulated blood was pumped into a spinning separation container. Red cells were packed by centrifugal force towards the outer edges of the container, and then the red cells exited the separation container. The lower density components, such as plasma, platelets, or WBCs were removed by plasma pump and entered the spinning collection container where platelets were packed by centrifugal force towards the outer edges of the container. The separated platelets remain packed in the container, while other constituents of blood were returned to the donor. At the end of the collection procedure, the platelet collection bag was shaken vigorously to detach the platelets from the wall of the bag and kept for 1 hour at room temperature to make it an even suspension. This whole procedure required 1.5-2.5 hours. The final volume of the apheresis-PC ranged from 200-300 ml.

### Quality assessment of platelets

The quality assessment of single donor platelets (apheresis-PC) and random donor platelets [platelet rich plasma-platelet concentrate (PRP-PC) and buffy-coat poor-platelet concentrate (BC-PC)] were done. A total of 146 platelet units, (PRP-PC, BC-PC and apheresis-PC: 64, 62 and 20 units respectively) were selected randomly and tested for the following parameters:

1. Platelet concentrates volume. 2. Swirling. 3. Platelet counts per bag. 4. WBC counts per bag. 5. pH changes.

Sample collection: 2-3 ml of sample from platelet concentrates either SDP or RDP was collected aseptically in a plain vial.

#### Platelet concentrate volume

The volume was determined by subtracting the weight of the empty bag from that of full bag. To convert weight to volume, the resultant weight was divided by 1.03 specific gravity of PRP-PC and 1.06 specific gravity of BC-PC.

$$\mathrm{Volume}\;\mathrm{of}\;\mathrm{the}\;\mathrm{concentrate}\;\left(\mathrm{ml}\right)\;=\;\frac{\mathrm{Wt}.\;\mathrm{of}\;\mathrm{the}\;\mathrm{full}\;\mathrm{bag}\;-\;\mathrm{Wt}.\;\mathrm{of}\;\mathrm{empty}\;\mathrm{bag}}{\mathrm{Specific}\;\mathrm{gravity}}$$

#### Swirling

The swirling was evaluated by examining the units against light and scored as:

- Score 0: Homogen turbid and is not changed with pressure.
- Score 1: Homogen swirling only in some part of the bag and is not clear.
- Score 2: Clear homogenic swirling in all part of the bag.
- Score 3: Very clear homogen swirling in all part of the bag.

#### Platelet count

The platelet count in the bag was done either manually on a Neubauer counting chamber or automated cell counter (MS 4 Melet Schloesing Laboratories, France).

Method for manual platelet counting: 1:20 dilution was made by adding a 50 µl sample of platelet concentrate into 950 µl of lysing fluid (1% aqueous ammonium oxalate) and kept for 10 to 15 minutes at room temperature with intermittent mixing. The Neubauer counting chamber was charged with diluted sample and kept for another 20 minutes in a moist Petri dish. Platelet counting was done in 5 small squares of the central square of the chamber under microscope (× 40 objective).

Calculation:

$$\mathrm{Platelets}\;\mathrm{count}\;\left(\mathrm{per}\;\mathrm{ml}\right)\;=\;\frac{\mathrm{No}.\;\mathrm{of}\;\mathrm{cells}\;\mathrm{counted}}{\mathrm{Volume}\;\mathrm{of}\;\mathrm{the}\;\mathrm{chamber}\;\left(µl\right)}\;\times \;\mathrm{dilution}\;\times \;1,000$$

$$=\frac{N}{0.02}\;\times \;20\;\times \;10^{3}$$

$$=\;N\;\times \;10^{6}$$

The platelet count per bag was calculated by: Platelet count per bag = N x 10^6^ x platelet concentrate volume

#### WBC count

WBC count in the bag was performed manually using a Neubauer counting chamber and WBC diluting Turk’s fluid [lysing fluid] (Gentian violet and 2% glacial acetic acid), Ranbaxy, India.

Method: 1: 2 dilution was made by adding an equal amount of whole blood (500 µl) into lysing fluid (500µl), which was mixed for 2 minutes and the Neubauer counting chamber was charged. Charged chamber was left for a further 2 minutes to settle the WBCs and counting was done in four large squares of the chamber under microscope (x 40 objective).

Calculation:

$$\mathrm{WBC}\;\mathrm{count}\;\left(\mathrm{per}\;\mathrm{ml}\right)\;=\;\frac{\mathrm{No}.\;\mathrm{of}\;\mathrm{cells}\;\mathrm{counted}}{\mathrm{Volume}\;\mathrm{of}\;\mathrm{the}\;\mathrm{chamber}\;\left(µl\right)}\;\times \;\mathrm{dilution}\;\times \;1,000$$

$$=\frac{N}{0.04}\;\times \;2\;\times \;10^{3}$$

= N × 5,000

The WBC count/bag was calculated by: WBC count/bag = N × 5,000 × platelet concentrate volume

#### pH evaluation

The pH was evaluated at the end of the maximum day of storage and pH indicator solution (Renkem, Ranbaxy, New Delhi), was used for pH assessment.

#### Method

1:50 dilution was made by adding 25 µl of indicator solution with 1250 µl of platelet concentrate in 75 × 10 mm glass tube. The color of the tube was compared against the colored strip provided with the indicator solution.

### Statistical analysis

All data were expressed as mean ± SD. We performed statistical comparison by using ‘t’-test for multiple groups. A probability of *P* < 0.05 (two-sided) was used to reject null hypothesis.

## Results

In this prospective study, quality of the platelet concentrates was assessed by observing swirling, volume, platelet count/bag, WBC count/bag and pH. Of these, swirling, volume, and platelet counts were evaluated in every unit, but WBC counts and pH were assessed in selected units.

Quality assessment of platelet concentrates

### Swirling

Swirling was observed in an individual unit and scored as score 1-3 according to subjective observation. Swirling with score 3 was observed in 79.7%, 83.9% and 90% of units while score 2 swirling was noticed in 20.3%, 16.1% and 10% of PRP-PC, BC-PC and apheresis-PC units respectively. No unit had score 3 swirling. Statistically no significant difference between PRP-PC and BC-PC units with regard to swirling was observed. Apheresis-PC units showed better swirling than PRP-PC and BC-PC units; this difference was statistically significant (*P* < 0.01).

### Volume (ml)

The volume of individual units was calculated and analyzed. The mean volume of the PRP-PC unit was 62.30±22.68 (mean ± SD) and ranged from 22-135 ml. The mean volume of BC-PC was 68.81± 22.95 ml (mean ± SD) and ranged from 32-133 ml, and the mean volume of apheresis PC unit was 214.05±9.91 ml (mean ±SD) and ranged from 200-251 ml.

The number of units (%) of PRP-PC, BC-PC and apheresis-PC meeting the desired quality control criteria of volume were analyzed. Of 64 PRP-PC units, 3 (4.6%) had volume < 40 ml while 13 (20%) units had volume of >70 ml. Thus 48 (75.4%) units met the desired quality control criteria of volume.[3] Of 62 BC-PC units, 32 (51.16%) had volume < 70 ml and 13 (21%) had volume >90 ml. Thus 17 (27.4%) units met the desired quality control criteria volume. A statistically significant difference was observed on comparing the maximum number of PRP-PC units with BC-PC units which met the desired quality control criteria of volume per unit [48 (75.4%) vs 17 (27.4%)]. All units of apheresis-PC (100%) met the recommended quality control parameter of volume.[3]

### Platelet count per unit

Platelet count of individual units was calculated and analyzed. Sixty-four PRP-PC units were assessed and the mean platelet count was 7.60±2.97 × 10^10^ (mean ± SD) per unit and ranged from 3.2-16.2 × 10^10^ per unit. The mean platelet count of 62 BC-PC units was 7.30±2.98 × 10^10^ (mean ± SD) per unit and ranged from 0.6 – 16.4 × 10^10^ per unit. The mean platelet count of apheresis-PC units was 4.13±1.32 × 10^11^ (mean ± SD) per unit and ranged from 1.22 – 8.9 × 10^11^ per unit. [Table 2]

**Table 2:** Platelet count per unit of platelet concentrates

Platelet count per unit	PRP-PC*	BC-PC*	Apheresis-PC
	(n = 64)	(n = 62)	(n = 20)
PRP-PC and BC PC (× 1010)			
Apheresis-PC (× 1011)			
Mean + SD	7.60 + 2.97	7.30 + 2.98	4.13±1.32
Range	3.2-16.2	0.6-16.4	1.22-8.9

*P* value-PRP-PC* vs. BC-PC*- not significant

The number of units (%) of PRP-PC, BC-PC and apheresis-PC meeting the desired quality control criteria of platelet count per unit were analyzed.[4] Seventy-eight point two percent (50/64) of PRP-PC units and 83.9% (52/62) of BC-PC units had platelet counts >5.5 × 10^10^ per unit, while 21.8% (14/64) of PRP-PC units and 16.1% (10/62) of BC-PC units had a platelet count <5.5 × 10^10^. On statistical analysis, of those meeting and not meeting the desired requirements no significant difference was observed between PRP-PC and BC-PC units. Ninety percent (2/20) units of apheresis-PC met the desired quality control criteria for platelet count i.e., >3.0 × 10^11^ per unit. A highly significant statistical difference was observed between the PRP-PC vs. apheresis-PC (*P*< 0.01) and between BC-PC vs. apheresis-PC units (*P*< 0.01) with regard to the units meeting the desired quality control criteria for platelet count per unit.

### WBC contamination per unit

WBC count was done on fresh units at day zero and a total of 26 units were analyzed. Of these units, 10 each were PRP-PC and BC-PC and 6 units were apheresis-PC. The mean WBC contamination in PRP-PC unit (n = 10) was 4.05 ± 0.48 × 10^7^ (mean ± SD) per unit and ranged from 3.4 – 4.7 × 10^7^. In BC-PC unit (n = 10), WBC contamination was 2.08 ± 0.39 × 10^7^ (mean ± SD) per unit and ranged from 1.6 – 2.7 × 10^7^. On comparison, BC-PC units had less mean WBC contamination than PRP-PC per bag and the difference was statistically significant (*P*< 0.001). Of 20 Apheresis-PC units, 6 were tested for WBC contamination and the mean WBC count was 4.1±0.8 × 10^6^ per unit and ranged from 3.2-5.2x10^6^. The mean WBC contamination of apheresis-PC unit was compared with PRP-PC and BC-PC unit. It was found that apheresis-PC units had least WBC contamination per unit than BC-PC and PRP-PC units and the difference was statistically significant (*P*< 0.001) [Table 3].

**Table 3:** WBC contamination per unit

Platelet concentrates	WBC Count per unit	

	Mean ± SD	Range
PRP-PC* (n = 10) [×107 per unit]	4.05±0.48	3.4-4.77
BC-PC* (n = 10) [×107 per unit]	2.08±0.39	1.6-2.7
Apheresis-PC $ (n = 6) [×106 per unit]	4.8±0.8	3.2-5.2

*P* value-PRP-PC* vs. BC-PC*- statistically significant (*P*< 0.001); Apheresis-PC $ vs. PRP-PC*/BC-PC*-statistically significant (*P*< 0.001)

*P* value-PRP-PC^*^ vs. BC-PC^*^- statistically significant (*P* < 0.001); Apheresis-PC^$^ vs. PRP-PC^*^/BC-PC^*^-statistically significant (*P* < 0.001)

On the basis of these results it was concluded that apheresis-PC units had least WBC contamination followed by BC-PC units. PRP-PC units had maximum WBC contamination, although all three types of platelet concentrates fulfilled the recommended quality control criteria for WBC count.[3]

### pH changes

A total of 26 units were analyzed for pH changes at day zero which was also the day of issue of the unit. Out of these units, 10 each were PRP-PC and BC-PC and 6 units were apheresis-PC. The mean pH was 6.7±0.26 (mean ± SD) and ranged from 6.5-7.0 and no difference was observed among the three types of platelet concentrates.

### Scoring

Scoring was done on the basis of parameters (i.e., swirling, volume, platelet count, WBC count and pH changes) taken for quality control evaluation for platelet concentrate units. Score was given according to number of parameters fulfilled by each unit, for example, score 5 or 4 was given to those units which fulfilled 5 or 4 recommended quality control parameters etc. WBC counting and pH evaluation was done in selected units and all units which were tested fulfilled the desired quality control criteria.[4] We assumed that all units which were transfused, had WBC count and pH within the recommendation for quality control of platelet concentrates. [Table 4]

**Table 4:** Scoring of platelet concentrate units

Scoring	PRP-PC		BC-PC		Apheresis-PC		Total	
	
	n = 64	%age	n = 62	%age	n = 20	%age	n = 146	%age
5	34	53.1	12	19.4	18	90	64	43.9
4	17	26.5	38	61.4	-	-	55	37.7
3	11	17.2	6	9.6	2	10	19	13
2	2	3.2	6	9.6	-	-	8	5.4
1	-	-	-	-	-	-	-	-

## Discussion

The ability of transfused platelets to circulate and function is dependent on both the effect of the *ex-vivo* storage lesions that undermines platelet functionality and the status of the *in vivo* milieu of the transfused individual.[5,6] In hospitalized thrombocytopenic patients factors affecting platelet consumption may be so strong in their influence on platelet recovery and survival that they may outweigh the effects of in vitro platelet storage.[7,8] Nevertheless it has long been recognized that changes in platelets that occur during storage can contribute to poor platelet function and decreased post transfusion survival. Changes in platelets fall into three broadly defined categories: platelet activation, metabolic alterations and platelet senescence. Normal platelet senescence is most likely a relatively minor component of the storage lesion. PCs that have been gently prepared and then immediately transfused without a significant storage interval (within 24-48 hours of donation) have uniformly high recovery, good survival and preserved function.[9] The storage of platelets was found to be associated with higher levels of platelet activation (i.e., about 10% of the release was associated with the preparation and about 30% with the subsequent storage period).[10]

Although 5-day storage of platelet concentrates is generally practiced in the developed countries, in our department platelets are usually issued within 24-48 hours of preparation to minimize chances of bacterial contamination and preserve function.

### The maximum shelf life of platelets in our department is 72 hours.

Quality assessment of platelet concentrates is an important step to evaluate *ex-vivo* functional viability of platelet concentrates and post transfusion recovery and survival in recipients. Various parameters are used for routine *ex-vivo* quality assessment of platelet concentrates such as swirling, volume, platelet count, WBC count and pH. Although other parameters are also used such as, measurements of ATP, membrane glycoprotein levels (P-selectin, GP Ib, GP IIb-IIIa) etc., these tests are cumbersome, not well standardized and difficult to perform on every PC unit in a routine setting. The *in vivo* viability of a transfused platelet product is determined by the percentage of the transfused platelets recovered in the recipient’s circulation immediately after transfusion (% recovery) and by the life span in circulation of these recovered platelets (survival).[11]

Commonly used methods for evaluation of *in vivo* viability (i.e., therapeutic efficacy) of platelets are corrected count increment (CCI) and percentage recovery (PR).

In our study, platelet concentrates were prepared by three different methods-viz, PRP-PC, BC-PC and apheresis-PC. *In vitro* quality of platelet concentrates was assessed by observing swirling, volume of PC, platelet count/unit, WBC count/unit and pH. A total of 146 PCs (PRP-PC:64, BC-PC:62 and apheresis-PC:20) were enrolled randomly.

### Quality assessment of platelet concentrates

The platelet concentrates were stored at 20-24° C with continuous agitation as recommended, until the time of issue.

Swirling: Evaluation of swirling is a simple noninvasive procedure that can performed by visual inspection and is useful for routine quality control of each individual PC on a large scale. Visual inspection of swirling correlates with platelet morphology; the presence of swirling indicates discoid morphology and absence is indicative of spherical morphology. Score 3 swirling was observed in 79.7%, 83.9% and 90% of units while score 2 swirling was noticed in 20.3%, 16.1% and 10% of PRP-PC, BC-PC and apheresis-PC units respectively. No unit had score 1 swirling. A study by Bertolini[12] reported that fresh PCs have positive swirling in 83% of units and negative in only 2%, the rest having intermediate swirling. After 5 days of storage, the proportion of PCs with positive swirling decreased to 65% and Bertolini concluded that this drop of swirling could be due to lesions that are known to occur during platelet preservation. Hence in fresh units the results of swirling in the present study were comparable to reported data.

Volume: Platelets prepared from whole blood collections or plateletpheresis are stored in donor plasma, which serves as a buffering agent. PCs from RDPs are typically suspended in 40 to 70 ml plasma to maintain pH. The major reason for using this volume range was based on early studies with PCs stored in first generation PVC containers. Because of the insufficient permeability of these containers to oxygen, there was a risk of a drop in the pH in the PCs from anaerobic conditions and elevated lactic acid production. The platelet-suspending volume was, therefore, maximized to increase buffering capacity while maintaining as little volume as possible, to minimize the risk of volume overload of the recipient’s circulatory system.[13] With the advent of new second generation highly oxygen permeable containers, there is little risk of exhausting the plasma-buffering capacity, since platelet energy metabolism is predominantly aerobic. Under these conditions, there is relatively little information on how much plasma is required to maintain viability. Storage of PCs with reduced plasma volumes has the advantages of less volume load to the recipient and of saving the plasma, which may be used for other purposes. Murphy *et al*.[14] indicated that PCs stored at a reduced volume of 30 ml for 5 days in a polyolefin container (PL-732, Fenwal, Deerfield, IL) had reduced post transfusion percentage recoveries compared to those PCs stored at a volume of 50 ml or more. Adams *et al*.[15] have suggested the PCs may be stored for 5 days with a volume as low as 30 ml without significant changes in vitro platelet characteristics that are believed to reflect platelet viability and hemostatic function.

In the present study, the mean volume of PRP-PC, BC-PC and apheresis-PC was 62.30± 22.68 ml, 68.81± 22.95 ml and 214.05± 9.91 ml and ranged from 22-135 ml and 32-133 ml and 200-251 ml respectively. All units of apheresis-PC (100%) had the desired volume, while 75.4% (48/64) and 27.4% (17/64) of PRP-PC and BC-PC units respectively fulfilled the quality control criteria of volume and the difference was significant between these 2 groups (*P* < 0.05).[3] Four point six percent (3/64) of PRP-PC units and 51.6% (32/62) of BC-PC units had volume less than recommended in quality control parameters. Though 51.6% (32/62) of BC-PC units had volume less than 70 ml, but certainly higher than 40 ml, and various studies[15,16] have shown that a volume >40 ml maintained the pH >6.2. Each platelet function and viability was preserved after 5 days of storage at room temperature with continuous agitation. Twenty percent (13/64) of PRP-PC units and 21% (13/62) of BC-PC units had volume more than desired for quality control of volume but higher volume does not have any deleterious effect on platelet function and maintains the pH throughout the storage period by its buffering action. In case of PRP-PC, 2 of the units had volume less than 40 ml (22 ml and 32 ml) and two units of BC-PC also had volume less than 40 ml (32 ml and 34 ml) and this volume would be suboptimal for storage. In a study by Hirosue *et al*.[16] there was no significant difference in the mean volume of either PRP-PC and BC-PC and it was 41±2 ml of both types of PCs. The mean value of our PRP-PCs and BC-PCs are comparable to those reported by Fijnheer *et al*.[17] However, the range and standard deviation (SD) in our units is wide, thus more standardization is required in their preparation.

The units which were tested for quality assessment in our study were fresh (<24 hours) and collected in Teruflex XT - 612 (PVC + di-2 ethylhexyl phthalate), TERUMO PENPOL (India), platelet storage bags, which can store platelets for a maximum of 5 days. A study done by Synder *et al*.[10] where various types of plastic platelet storage bags were used, found that the platelet concentrates stored in Teruflex XT –612 (Terumo) at 22°C with continuous agitation had pH >7.0 and a platelet count >5.5 × 10^10^/unit at the end of day 5.

PCs with volumes ≥35 ml showed high degree of similarity in both in vivo and in vitro activities of platelets. Significant reduction in viability associated with increased lactate production was found in PCs which had volume ≤30 ml. Several previous studies have shown that increased lactate levels correlate with reduced post transfusion viability.[18,19] A probable cause for the reduction in viability associated with increased lactate production may be that a reduction in the suspending volume results in increased container surface-to-PC volume ratio, which may lead to more frequent platelet container wall interactions, with a risk of increased platelet activation or stimulation. Studies by Bode *et al*.[20] implied that the container surface area-to-volume ratio is important for platelet activation during storage. It has been shown that reducing the container surface area from 7 cm^2^ to 4 cm^2^ leads to less activation as demonstrated by less lactate output and improved maintenance of platelet morphological and functional integrity during storage.

Another factor that could potentially causes loss of viability by storage of PCs in volume below 35 ml is the increased risk of a drop in pH resulting from the reduction in the buffering capacity of the suspending medium.

### Platelet count

During the preparation of PCs there is deterioration of platelet function manifested by abnormal shape changes, aggregation and secretory response. The main cause of deterioration of platelet function during preparation is lesions associated with the preparative manipulation and storage. In a study reported by Bode *et al*.,[4] in BC-PC units after 48 hours of storage the shape change, aggregation, and secretory responses were at the same levels as those in PRP-PCs units immediately after preparation.[4] However, the difference between PRP-PC and BC-PC units became less significant after 4 days of storage. The results of this study are consistent with another study in which after 5 days of storage the author did not find any difference in either type of PCs regarding morphological changes as well as in vivo survival.[3]

In the present study, the mean platelet count of PRP-PC, BC-PC and apheresis-PC was 7.6± 2.97 × 10^10^/unit, 7.3± 2.98 × 10^10^/unit and 4.13± 1.32 × 10^11^/unit and ranged from 3.2-16.2 × 10^10^/unit, 0.6-16.4 × 10^10^/unit and 1.22-8.9 × 10^11^/unit respectively. The mean platelet count of PRP-PC and BC-PC was comparable and statistically no significant difference was observed. The number of units (%) of PRP-PC, BC-PC and apheresis-PC meeting the desired quality control criteria of platelet count were also analyzed.[3] Seventy-eight point two percent (50/64) of PRP-PC and 83.9% (52/62) of BC-PC units had platelet counts >5.5 × 10^10^/unit, while 21.8% (14/64) of PRP-PC units and 16.1% (10/62) of BC-PC units had a platelet count <5.5 × 10^10^/unit and on comparison between these two no significant difference was observed. Ninety percent (18/20) of apheresis-PC units met desired quality control criteria for platelet counts i.e. >3 × 10^11^/unit and a highly statistical significant difference was observed between apheresis-PC vs. PRP-PC/BC-PC units (*P* < 0.01) with regard to the units meeting the desired quality control criteria for platelet count/units.

Although a higher number of apheresis-PC units met the desired quality control for platelet count, the other two types of PCs fulfilled the desired quality control norm of platelet count (i.e. 75% of tested units should met the recommended quality control criteria of platelet count).

Fijnheer *et al*.[17] reported 15% higher platelet yield in PRP-PC than BC-PC units. Hirosue *et al*.[16] also reported higher platelet count in PRP-PC units than BC-PC. Murphy *et al*.[14,21] found that the platelet recovery was higher in patients who received PRP-PC (60-70%) than those with BC-PC (40-60%) transfusion. No such difference was observed in the present study and the mean platelet count/unit in both types of platelet concentrates was comparable and statistically no significant difference was observed. [Table 5]

**Table 5:** Comparisons of volume, platelet counts, and WBC counts of PCs with study groups

Study groups	Volume (ml)		Platelet count/unit (× 1010)		WBC count/units (× 107)	

	PRP-PC	BC-PC	PRP-PC	BC-PC	PRP-PC	BC-PC
	(mean + SD)	(mean + SD)	(mean + SD)	(mean + SD)	(mean + SD)	(mean + SD)
Hiroshue *et al*.[16]	41+2	41+2	7.62+1.8	6.12+1.51	3+1	<1
Fijnheer *et al*.[17]	67.8+4.0	56.2+6.7	7.0+1.0	5.9+12.2	1.9+1.5	0.07+0.08
Pieterz *et al*.[22]	70+29	101+5	7.0+2.9	8.9+3.4	-	-
Present study	62.3+22.68	68.81+22.95	7.6+2.97	7.3+2.98	4.05+0.48	2.08+0.39
	(22-135)*	(32-133)*	(3.2-16.2)*	(0.6-16.4)*	(3.4-4.7)*	(1.6-2.7)*

* range

Bertolini *et al*.[23] also reported lower platelet yield in BC-PC units as compared to PRP-PC units however platelet count increments resulting after transfusion of BC-PC and PRP-PC units were not significantly different.

### WBC Count

WBCs in PC have a detrimental effect on the storage medium, resulting in a significant drop in pH, increase in glucose consumption, lactic acid production and LDH release during storage. As a result, in the PCs with high concentration of leukocytes, the platelet condition up to 5 days of storage was also significantly affected, as reflected by a high excretion of β-TG, loss of platelet nucleotides, decreased ability to incorporate[3] H-adenosine and poor platelet morphology.[24] In addition to these, transfused passenger leukocytes during platelet therapy may be associated with a variety of adverse effects, including alloimmunization to leukocyte antigens, febrile non-hemolytic transfusion reaction (FNHTR), refractoriness to platelet transfusion, severe pulmonary dysfunction, graft versus host disease (GVHD), the transmission of cytomegalovirus (CMV) and immune modulation.[12] Platelet concentrates made from individual units of fresh whole blood may contain from 0.5 × 10^8^ to 2.5 × 10^8^ WBC/unit.[25] Apheresis platelets that have been harvested using old instruments may contain up to 5 × 10^9^ leukocytes, while apheresis platelets obtained using more recently available instruments contain from 10^6^ to 5 × 10^8^ WBCs.[26] In this study WBC counting was done on fresh units at day zero and a total of 26 units were analyzed. The mean WBC count in PRP-PC (n = 10), BC-PC (n = 10) and apheresis-PC (n = 6) units was 4.05 ± 0.48 × 10^7^/unit, 2.08 ± 0.39 × 10^7^/unit and 4.8 ± 0.8 × 10^6^/unit and ranged from 3.4-4.77 × 10^7^/unit, 1.6-2.7 × 10^7^/unit and 3.2-5.2 × 10^6^/unit respectively. BC-PC units which were tested had significantly less WBC count/unit than PRP-PC units (*P* < 0.001). Hirosue *et al*.[16] compared components prepared by the PRP-PC and BC-PC methods and had WBC count/unit of 3 ± 1 × 10^7^/unit and < 1 × 10^7^/unit respectively. The results of Fijnheer *et al*.[17] were also similar and reported WBC contamination/unit in PRP-PC and BC-PC was 1.9 ± 1.5 × 10^7^ and 0.07 ± 0.08 × 10^7^ respectively. The results of both the above studies had similar results regarding WBC count/unit as compared to present study, thus highlighting the advantage of BC-PC when compared to PRP-PC as regards to leukocyte contamination [Table 5].

Burgstaler *et al*.[27] compared two apheresis systems and reported mean WBC count of 0.67 × 10^6^/units, in apheresis-PC units which were collected by CS 3000 plus. Seagraves *et al*.[28] using manual counting techniques (unspecified) and a mean WBC of 2.4 ± 0.7 × 10^6^/unit was reported. We found higher mean WBC count/unit i.e. 4.8 ± 0.8 × 10^6^ , as compared to these two studies, but the counts were below the permissible limit (i.e. < 5 × 10^6^/unit).

Our results of WBC count/unit reveal that apheresis-PC unit had least WBC count/unit followed BC-PC. PRP-PC unit had maximum WBC contamination although all three types of PCs fulfilled the recommended Q.C. criteria for WBC count.[3] An upper limit of 10^7^ leukocytes per PC provides good storage conditions and contributes to prevent HLA alloimmunization in recipients. Also with PC derived from PRP, the leukocyte contamination can be diminished if the handling of the bags and the transfer of the PRP are performed carefully.[29]

### pH change

The pH decreases during storage depending on the stabilizer in plastic platelet storage bags and storage conditions used. Increased platelet glycolysis resulting in a fall in pH to levels approaching 6.0 in PC stored in plasma is associated with substantial loss of viability.[10] The majority of fresh, un-stimulated platelets are discoid with few projections. In the early observations of PCs stored at 20-24° C, a gradual disc-to-sphere transformation was seen during storage. Some of these changes are reversible with incubation at 37° C in fresh plasma. Qualitatively similar changes occur during PC storage, but in first generation containers major additional variable is pH fall. If pH does not fall to less than 6.8, platelet volume decreases by approximately 10% during three days. However, if pH falls below this level, there is a progressive rise in platelet volume and decrease in density suggesting swelling due to influx of extracellular fluid. The swelling begins at pH of 6.8 and reaches its maximum at a pH of 6.0, at which point platelet volume is increased almost two-fold. At the same time, there is an accelerated rate of disc-to-sphere transformation so that only swollen spheres are seen if pH reaches 5.7 to 5.9. These changes are almost entirely reversible if pH stays above 6.1, but they are not reversible if pH falls below 6.1. These morphological observations correlate well with the results of viability in vivo.[21]

In the present study a total of 26 units were analyzed for pH changes. Out of these units, 10 each were PRP-PC and BC-PC and 6 units were apheresis-PC. Their mean pH was 6.7 ± 0.26 (mean + SD) and ranged from 6.5 – 7.0 and no difference was observed among all three types of platelet concentrate. Synder *et al*.[30] reported that at 5 days of storage, the pH of all PC was >7.0 and platelet count was above 5.5 × 10^10^ per bag (except for the PL-732) with the 6 rpm vertical rotator. Banna *et al*.[31] reported that at pH 6.85, vertical agitation platelet damage was minimal up to 12 hours, but increased from 36 hours after the pH increased to 7.3 to 7.4 and these results indicate that vigorous agitation is not as injurious at low pH. However in the present study since platelets were issued within 24 hours of preparation, the pH on further storage was not assessed.

### Scoring

In this study, scoring was done on the basis of parameters (i.e., swirling, volume, platelet count, WBC count and pH) used for quality control evaluation of platelet concentrates. A total of 146 units were evaluated and of these 43.9% (64/146) had score 5 i.e. fulfilled all 5 parameters of quality control and 37.7% (55/146) units had score 4. On individual analysis, maximum number of apheresis-PC units had score 5 i.e. 90% (2/20) and followed by PRP-PC units i.e. 53.1% (34/64), but only 19.4% (12/62) of BC-PC units fulfilled all 5 desired quality control criteria. This overall difference in score 5 and 4 was basically due to variation in volume in BC-PC units and only 27.4% (17/62) managed to fulfill the desired quality control criteria of volume, although as already discussed the units had more than the minimum limit of 40 ml. Nevertheless, maximum number of platelet concentrate units, which were evaluated for quality assessment had score 5 and 4 i.e. 81.6% (119/146). Thus in our study majority of the prepared units were of the desired quality.

## Conclusion

PRP-PC and BC-PC units were comparable in terms of swirling, platelet count per unit and pH. As expected, we found WBC contamination to be less in BC-PC than PRP-PC units. Variation in volume was more in BC-PC than PRP-PC units and this suggests that further standardization is required for preparation of BC-PC. As compared to the above two platelet concentrates, all the units of apheresis-PC fulfilled the desired quality control criteria of volume. Apheresis-PC units showed better swirling and platelet count than PRP-PCs and BC-PCs. All the platelet concentrate units had pH well above the recommended norm.

Our study suggests though that the apheresis platelets are superior to PRP-PC and BC-PC in terms of platelet counts.

Thus, in developing countries apheresis platelets, because of their high cost and more technical expertise required may be recommended only in selected patients either when PRP-PC and BC-PC in adequate doses are not available in the inventory, or when HLA-matched platelet transfusions are indicated.
